# Mapping urban socioeconomic inequalities in developing countries through Facebook advertising data

**DOI:** 10.3389/fdata.2022.1006352

**Published:** 2022-11-21

**Authors:** Simone Piaggesi, Serena Giurgola, Márton Karsai, Yelena Mejova, André Panisson, Michele Tizzoni

**Affiliations:** ^1^Institute for Scientific Interchange Foundation, Turin, Italy; ^2^Dipartimento di Informatica - Scienza e Ingegneria, Alma Mater Studiorum University of Bologna, Bologna, Italy; ^3^Department of Network and Data Science, Central European University, Wien, Austria; ^4^Alfréd Rényi Institute of Mathematics, Budapest, Hungary; ^5^CENTAI Institute, Turin, Italy; ^6^Department of Sociology and Social Research, University of Trento, Trento, Italy

**Keywords:** poverty mapping, advertising data, social networks, bias, urban development

## Abstract

Ending poverty in all its forms everywhere is the number one Sustainable Development Goal of the UN 2030 Agenda. To monitor the progress toward such an ambitious target, reliable, up-to-date and fine-grained measurements of socioeconomic indicators are necessary. When it comes to socioeconomic development, novel digital traces can provide a complementary data source to overcome the limits of traditional data collection methods, which are often not regularly updated and lack adequate spatial resolution. In this study, we collect publicly available and anonymous advertising audience estimates from Facebook to predict socioeconomic conditions of urban residents, at a fine spatial granularity, in four large urban areas: Atlanta (USA), Bogotá (Colombia), Santiago (Chile), and Casablanca (Morocco). We find that behavioral attributes inferred from the Facebook marketing platform can accurately map the socioeconomic status of residential areas within cities, and that predictive performance is comparable in both high and low-resource settings. Our work provides additional evidence of the value of social advertising media data to measure human development and it also shows the limitations in generalizing the use of these data to make predictions across countries.

## 1. Introduction

Reduction of poverty is the number one goal of the United Nations, as defined in their Sustainable Development Goals (SDG)^1^. However, in order to address this age-old condition, copious amounts of data need to be collected, and often it is in the places most at risk that it is most difficult to perform surveys. In 2020, the World Bank has admitted that surveying and face-to-face interviewing have been hindered by the COVID-19 epidemic and the resulting distancing measures^2^. Technology-assisted surveys, including phone-based interviews, are becoming invaluable tools. However, many countries lack the resources to run such data collection exercises, and monitor the socioeconomic status (SES) indicators only every several years, some only every decade.

Cities are remaining at the center of these developments, as urban populations grow rapidly, and are projected to do so in the near future (Department for International Development (DFID), 2001; Jha, 2020). Urbanization contributes to global economic growth, provides opportunities for millions of people, attracts investors and entrepreneurs, and offers much needed services (Baker, 2008). However, it also suffers from maladies spanning overcrowding and inadequate housing, lack of social networks, stark inequality, crime, and violence. In some cases, undocumented residents forego the benefits of urbanization and miss out on government assistance, such as during the COVID-19 pandemic, when the joblessness disproportionally affected non-white and female workforce (Urban Institute, 2020). In such dynamic circumstances, the well-being of urban residents often hinges on the inclusivity, infrastructure, and other measures taken by their urban governance systems (The World Bank, 2020).

To keep track of the urban SES indicators, recent research has turned to alternative data sources, including the daytime (Jean et al., 2016; Engstrom et al., 2017; Watmough et al., 2019) and nighttime satellite imagery (Chen and Nordhaus, 2011; Mellander et al., 2015), mobile phone Call Detail Records (CDRs) (Fernando et al., 2018), and even crowd-annotated information from OpenStreetMap (Tingzon et al., 2019). However, even these data sources often suffer from being out of date and not easily available to the research community. In this paper, we use a resource which has been gaining attention in the demographic domain: the advertising platform of the largest social network, Facebook, accessible *via* the Facebook Graph API^3^. In particular, Facebook provides a way to gauge the reach of any advertising campaign by providing the number of daily or monthly active users (MAU) that certain constrains would reach. For instance, it is possible to target Facebook users by their gender, age, sets of interests, location (down to a circle of 1 km radius), and many other attributes^4^. These estimates provide a “view” of the billions of Facebook users without jeopardizing the privacy of any individual user, and they can be obtained automatically, without running the actual ads. Recently, Facebook Ads audience estimates have been used to track the prevalence of obesity and diabetes (Araujo et al., 2017), crime rates (Fatehkia et al., 2019), cultural assimilation (Stewart et al., 2019) and mass migration (Palotti et al., 2020). Socioeconomic development across the Indian states has been tracked using the gender disparities in the access to Facebook (Mejova et al., 2018), and in combination with satellite imagery it has been applied to Philippines and India for tracking Demographic and Health Survey Wealth Index (Fatehkia et al., 2020a). However, it is still unclear how applicable this information is at a fine granularity, and whether adoption rates of Facebook in the population would render Facebook too sparse for developing urban regions.

In this paper, we seek to fill this gap by answering the following research questions:

- **RQ1**: can Facebook advertising audience estimates provide insights into the socioeconomic conditions of populations at a high spatial granularity, namely at the level of urban subdivisions?
- **RQ2**: is the performance of a predictive model at such spatial resolution comparable across cities in high and low-income economies?
- **RQ3**: what Facebook users' attributes are most predictive of socioeconomic status within urban areas?

To this aim, we use Facebook audience estimates to predict SES of the districts of large urban areas: Atlanta (USA), Santiago (Chile), Bogotá (Colombia), and Casablanca (Morocco). We choose cities in both developed an developing countries, to gauge the effectiveness of our approach in different settings characterized by middle-low to high income economies. Through the Facebook marketing API, we measure the spatial distribution of monthly active users matching a wide range of targeting options, including demographics, and behaviors, within each city. We then use such estimates to train a Random Forest classifier to predict a binary SES classification: high vs. low SES. Due to varying data availability, we consider different indicators as a proxy for SES in the four cities, either average annual household income or poverty rate. All indicators, although measured differently, are related to household income. We test our model's performance against varying threshold definitions of high SES and low SES, and we compare results with different train-test splits, both in the same city and across cities.

Our results demonstrate that Facebook audience estimates can provide insights into the spatial patterns of wealth and poverty in urban areas, with good and comparable predictive performance between developed and developing countries. Furthermore, we also highlight limitations in the transferability of a SES classifier trained on a source city and exported to a different target, as model's performance varies significantly depending on the specific source and target cities.

## 2. Related work

The Data Revolution has made available not only existing records in a digitized form, but it has spurred an interest in alternative sources of data (Alburez-Gutierrez et al., 2019; Weber et al., 2021). Below, we summarize the latest efforts in using alternative digital signals, and social media in particular, in order to track SES.

The socioeconomic status (SES) of individuals is a complex character, which depends not only on one's economic capacities but also on the social and cultural position of the ego in the larger society. Quantifying such a convoluted character of a person is a very difficult if not impossible task (Oakes and Rossi, 2003; Vyas and Kumaranayake, 2006; Baumeister and Vohs, 2007). For this reason, data-driven studies usually approximate socioeconomic status by some easily observable variables, which sensitively reflect economic inequalities between people. Such indicators can be the income (Abitbol and Karsai, 2020) or the occupation (Abitbol et al., 2018) of an individual, or poverty level of one's residential neighborhood (Steele et al., 2017), just to mention a few examples. Meanwhile, for a meaningful analysis these indicators are needed to be available for larger populations as they commonly serve as ground truth data for supervised inference methods. For behavioral based inference individual indicators correlated with one's economic capacity are used, like bank (Leo et al., 2016a) and purchase records (Leo et al., 2016b). At the same time, location-based inference requires high-resolution income and demography maps typically recorded during census in developed countries (INSEE, 2019), or low-resolution poverty maps from under-developed countries are used for these purposes (Bank, 2021).

A variety of digital sources have been used to track development and SES, including satellite imagery (Elvidge et al., 2009; Piaggesi et al., 2019), mobile call log data (Blumenstock et al., 2015), and transport-related apps (Tan et al., 2019). Social media in particular has been used to provide deeper understanding of various population well-being indices. For instance, Resce and Maynard (2018) use Twitter to track the constituent issues comprising the Better Life Index (BLI) including income, employment, civic engagement, and health. Google Trends, a service providing an aggregated view of common Google search queries, has been used to track infectious and non-communicable diseases (Nuti et al., 2014), as well as unemployment and consumer confidence (Choi and Varian, 2012). Moreover, recent inference methods provide high-resolution estimates of poverty maps even in under-developed countries using combined data sources and validation on spatially aggregated levels (Lee and Braithwaite, 2020).

Compared to traditional data sources, social media offers several notable benefits. Due to the data being available in real time *via* Application Programming Interfaces (APIs) by the platforms, it can be used to provide rough estimates of ongoing phenomena, or help in “nowcasting” (di Bella et al., 2018). As official data often lags by as much as weeks or months, nowcasting using social media provides daily and even hourly information in volatile situations including disasters and ongoing events. Coverage is another benefit of social media—especially either of large platforms that have been widely adopted, or of smaller, more local platforms. For example, searches on Baidu have been used to estimate economic activity (Dong et al., 2017) and restaurant reviews to estimate socioeconomic attributes of urban neighborhoods (Dong et al., 2019) in China. However, the full datasets are usually not available for research, and the APIs provide a small glimpse into the vast user bases of major social media websites. Their advertising services, then, are an alternative route to learning about the users of large websites in a privacy-preserving fashion.

Facebook (and most other large websites) provides advertising services on its platform which allow potential advertisers to ascertain the size of the potential audience their campaign could reach. Along with the basic demographic, location, and technology use, it provides the advertisers to explore their audiences by a variety of interests and behaviors. Recently, demographers, sociologists, and other researchers have been using this information as a kind of “digital census.” Using Facebook Ads, a variety of demographic and economic indicators have been studied, such as the prevalence of obesity and diabetes (Araujo et al., 2017), crime rates (Fatehkia et al., 2019), cultural assimilation (Stewart et al., 2019) and mass migration (Palotti et al., 2020). However, the usefulness of such data may vary in different locales, especially compared to alternative sources of information. For instance, Fatehkia et al. (2020a) show that models trained on Facebook Ads data can predict the Demographic and Health Survey Wealth Index in Philippines about as well as those trained on satellite data. However, for India, satellite data performs better, possibly due to the lower penetration of Facebook. Especially useful may be the signals about the kind of technology that is available to the populations, such as mobile phones and network access (Fatehkia et al., 2020b). Recent work has also shown that ads audience data provided by Facebook suffers from inconsistency over time and poor coverage in sparsely-populated areas (Rama et al., 2020). Still, the same work has shown that it is possible to overcome some of these challenges, and to capture multiple dimensions of inequality between rural and urban municipalities in Italy (Rama et al., 2020). In this study, we examine the usefulness of Facebook Ads audience estimates both in developed and developing urban settings, and discuss the challenges and benefits it brings with regard to mapping the fine-grained SES levels.

## 3. Materials and methods

In this section, we describe the main data sources analyzed in our study and the methods used to predict the urban SES indicators.

### 3.1. Urban socioeconomic indicators

We began by collecting socioeconomic data in the cities of Atlanta, Santiago, Bogotá, and Casablanca from publicly available official sources. Table 1 summarizes the main characteristics of the data sets under study. The four data sets map the socioeconomic status of the neighborhoods in each city based on different indicators and at different spatial resolutions. We focus on a high spatial granularity, with the aim of capturing SES differences that are observed within the administrative boundaries of each city. In our study, these represent our main target that we aim to predict using advertising audience estimates as described below.

**Table 1:** Socioeconomic indicators used in the study and their corresponding data sources.

**City**	**Indicator**	**Unit**	**Year**	***N***
Atlanta, USA	Median household income	US Dollars (USD)	2019	40
Bogotá, Colombia	Socioeconomic strata	Levels 1 (low) - 6 (high)	2017	110
Santiago, Chile	Median household income	Chilean Pesos (CLP)	2012	36
Casablanca, Morocco	Multidimensional poverty rate	Population fraction	2014	17

The rightmost column indicates the number of data points, corresponding to the number of neighborhoods in each city.

**Figure 1:**
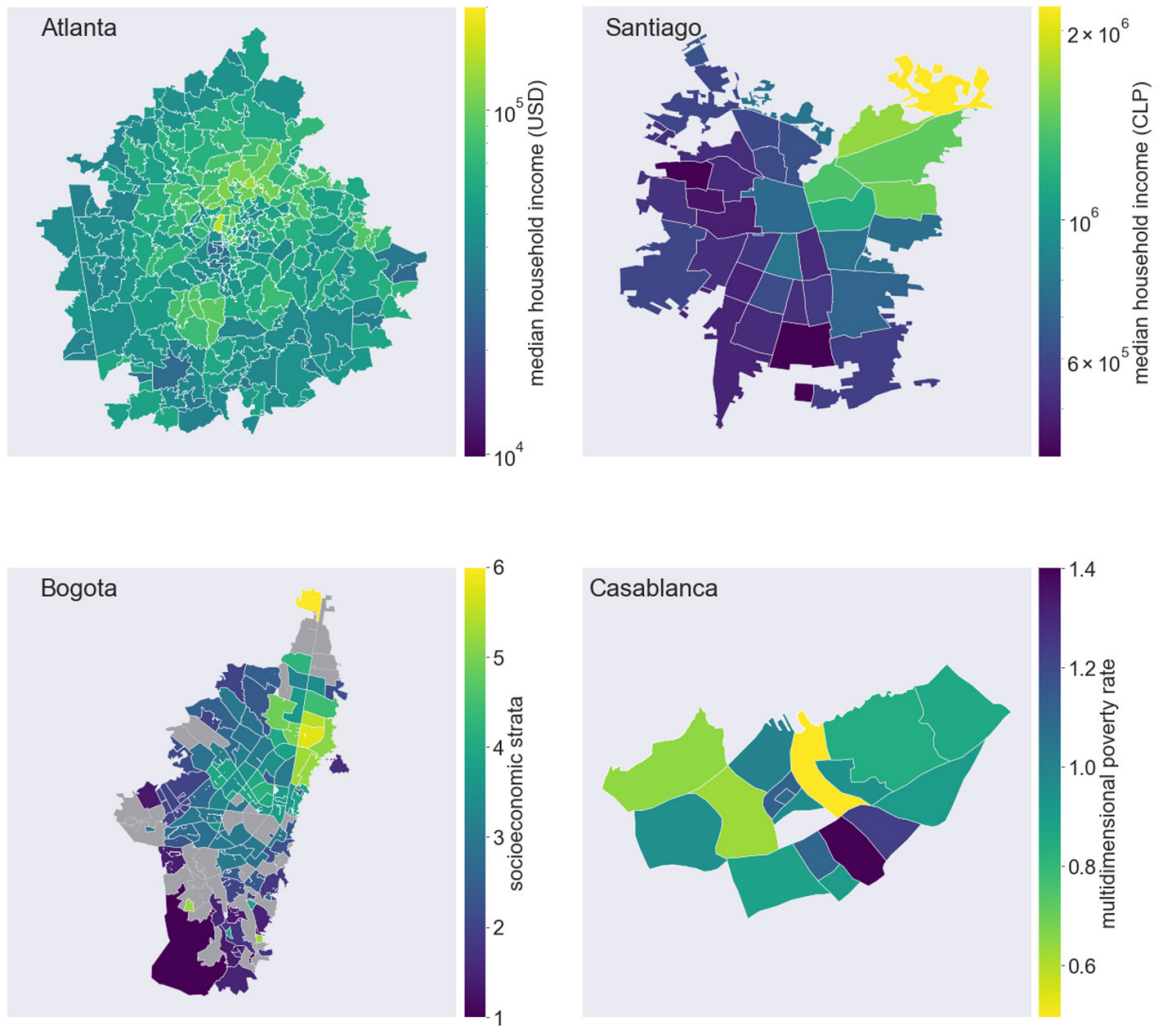
Spatial distributions of socioeconomic indicators. Choropleth maps display the values of socioeconomic indicators in the cities of Atlanta, Santiago, Bogotá and Casablanca. From top to bottom: median household income in Atlanta, USA, by zip code; median household income in Santiago, Chile, by comuna; socioeconomic strata in Bogotá, Colombia, by UPZ (missing data are colored in gray); multidimensional poverty rate, in Casablanca, Morocco, by arrondissement.

In the city of Atlanta, we considered the median household income in each of the 40 zip codes, as reported by the American Community Survey in 2019 (Census Reporter, 2021). In Santiago, Chile, we collected the 2012 median household income, in Chilean Pesos, reported by the Chilean Ministry of Transport and Communication (Programa de Vialidad y Transporte Urbano, 2012). We considered the urban part of the Santiago Metropolitan Area, that is composed of 36 municipalities, named *comunas*. Socioeconomic data for Bogotá are available from the *Secretaría Distritial de Planeación* and map the socioeconomic status of 110 neighborhoods (Unidades de Planeamiento Zonal or UPZ) on a discrete scale ranging from 1 (low income) to 6 (high income). Finally, in the 17 neighborhoods (*arrondissements*) of Casablanca, we considered the multidimensional poverty rate reported by the official census in 2014. The multidimensional poverty rate measures the population fraction living in poverty, according to the definition of the Higher Planning Commission of Morocco (Haut-Commissariat au Plan du Maroc, 2014) which takes into account 15 different dimensions, ranging from income to education, health and access to essential services. The spatial distribution of the socioeconomic indicators in the neighborhoods of Atlanta, Bogotá, Santiago and Casablanca is shown in Figure 1. As each location has a different standard for subdividing the neighborhoods, which usually aims to capture similar number of residents, resulting in different geo-resolution. In Section 3.3 we describe how these are further subdivided to perform fine-grained geographic data collection using the Facebook ads platform. *Arrondissements* in Casablanca correspond to the third administrative level of Morocco in the GADM database (University of California Berkeley, 2020). In the other cities, we consider subdivisions that are finer than the third GADM level.

In all cities, we can observe distinct geographic patterns with strong socioeconomic inequalities across districts. In Atlanta, the 2019 median household income ranged from 30,000 USD or less in Downtown and southern areas of the city, to 100,000 USD or more in the wealthiest residential areas of the North. Segregation patterns in Santiago follow a East-West divide, where the wealthiest neighborhoods are clustered in the Northeastern part of the city. A median household income above 1 million Chilean pesos is observed in only 5 out of 36 *comunas*. In Bogotá, higher socioeconomic strata of the population are concentrated in the neighborhood of Usaquén, in the North-East of the city. In Casablanca, the most deprived *arrondissements*, with a poverty rate above 1%, are found in the South-East suburbs of the city.

As the available SES indicators are different in the four cities, and they cannot be directly compared to each other, we set our study as a binary classification task: our goal is to predict which city districts belong to the low/high SES category. Low and high SES are defined based on the percentiles of the distributions of SES indicators. Below, we discuss how these scores are aggregated and binarized to define the high-SES/low-SES classification task. Identifying administrative areas that fall below a given income threshold is a task that has practical implications. Indeed, national and international assistance programs are always targeted at groups who fall below a given poverty threshold. More often, such programs identify eligible households among those who live in administrative units where the average income falls below a given threshold (Aiken et al., 2022; Smythe and Blumenstock, 2022).

### 3.2. Facebook advertising data

We collected Facebook Advertising audience estimates through the Facebook Marketing API using the Facebook business Python package^5^. Each query targets a geographic area of interest, either a city neighborhood or a circle with a fixed radius of 1 km. More details about the spatial definition of our queries are provided in Section 3.3. In each query, we request the count of Facebook users “who live there” (technically, by setting the location_type parameter to home), and who match a specific targeting option as described below. We constrain each query to select only Facebook users, although it is possible to query for users using other Facebook owned services, like Instagram. Given our interest in different countries, in this way we aim at an easier comparison and interpretation of results. Among the various advertising campaign types, we choose the “reach” option, which targets the “maximum number of people.” Finally, in the reply to our query, we save the number of Monthly Active Users (MAU), as done in previous studies, because it provides a more stable estimate with respect to the Daily Active Users (Rama et al., 2020).

Since each query does not usually return exactly the same response from the API (Rama et al., 2020), especially in less populated areas, we average all audience estimates over 3 identical queries, performed at different times, between January and September 2020. The Facebook marketing platform does not return estimates of MAU below the value of 1,000, and if a query targets a smaller number of users, then the API will return a value of 1,000—which is thus indistinguishable from 0. In our study, we replaced all query results equal to 1,000 with zeros and when a specific combination of target and location returned 1,000 users for all the 3 queries, we did not include it among the features of the predictive models.

We build on previous studies to choose attributes of Facebook users that are predictive of socioeconomic status (Fatehkia et al., 2018, 2020a). In particular, recent studies have shown that technology features such as the type and model of owned mobile devices, or the cell network used to access the Internet, are highly predictive of income (Fatehkia et al., 2020a). We extend our study to a wider range of features, querying audience estimates over a range of 35 attributes that pertain to demographics, culture, mobility and other behaviors. A complete list of attributes is reported in Table 2. The Monthly Active Users estimates returned by the Facebook marketing platform then were constrained by the query attributes. For instance, by querying for users who had a certain brand of cellphone, we could calculate the share of users in a locale who own such a cellphone (similarly for any other attribute). We theorize that such a statistic could be related to the local levels of SES.

**Table 2:** List of Facebook attributes considered as predictive features of SES.

**Category**	**Feature**	**Atlanta**	**Bogotá**	**Santiago**	**Casablanca**
Gender	Males	45 ± 8.6%	49 ± 0.7%	48 ± 1.7%	60 ± 1.4%
	Females	51 ± 9.4%	49 ± 1.0%	50 ± 1.2%	39 ± 1.0%
Marital status	Single	19 ± 10%	24 ± 0.7%	21 ± 0.9%	12 ± 0.3%
	Engaged	0.2 ± 0.9%	2.9 ± 0.2%	3.2 ± 1.1%	0.4 ± 0.3%
	Married	18 ± 11%	10 ± 0.4%	13 ± 1.7%	8.5 ± 0.4%
	Civil union	–	0.0 ± 0.0%	–	–
Education	High school grad	10 ± 6.7%	13 ± 0.6%	12 ± 1.5%	9.3 ± 0.2%
	College grad	34 ± 12%	31 ± 0.8%	30 ± 4.7%	22 ± 0.8%
Travel	Away from hometown	13 ± 7.5%	7.2 ± 0.4%	8.8 ± 2.0%	3.0 ± 0.2%
	Away from family	13 ± 7.4%	7.2 ± 0.4%	8.8 ± 2.0%	3.0 ± 0.2%
	Frequent international travelers	11 ± 6.3%	21 ± 0.9%	27 ± 4.1%	34 ± 1.0%
	Frequent Travelers	50 ± 11%	61 ± 1.1%	72 ± 1.2%	72 ± 0.6%
	Returned from travels 1 week ago	0.1 ± 0.5%	8.9 ± 0.4%	22 ± 1.3%	12 ± 0.2%
	Expats	12 ± 9.2%	6.8 ± 0.2%	14 ± 5.3%	5.5 ± 0.1%
Technology	iOS	27 ± 10%	7.3 ± 1.1%	10 ± 5.8%	5.7 ± 0.2%
	Android	24 ± 11%	68 ± 1.8%	64 ± 7.5%	73 ± 0.8%
	Mac	3.0 ± 3.9%	1.6 ± 0.5%	0.8 ± 1.1%	1.4 ± 0.4%
	Windows	2.7 ± 3.0%	4.4 ± 0.7%	5.1 ± 1.2%	0.3 ± 0.2%
	iPhone X	0.5 ± 1.2%	0.0 ± 0.1%	0.0 ± 0.0%	–
	iPhone 8	0.1 ± 0.6%	0.1 ± 0.2%	0.2 ± 0.4%	–
	iPhone 8 Plus	0.6 ± 1.3%	0.1 ± 0.1%	0.0 ± 0.0%	–
	Galaxy S8	0.0 ± 0.2%	–	0.0 ± 0.1%	–
	Galaxy S8+	0.0 ± 0.1%	–	0.0 ± 0.0%	–
	Galaxy S9	0.0 ± 0.2%	–	0.0 ± 0.0%	–
	Galaxy S9+	0.0 ± 0.2%	–	0.0 ± 0.0%	–
	Samsung Android	11 ± 7.2%	20 ± 1.1%	28 ± 2.1%	36 ± 0.5%
	Huawei	–	19 ± 1.0%	18 ± 2.9%	7.2 ± 0.2%
	Oppo	–	–	–	4.1 ± 0.2%
	Older devices	12 ± 8.6%	25 ± 0.9%	18 ± 1.5%	33 ± 0.6%
	Smartphone & Tablet	56 ± 13%	74 ± 3.0%	73 ± 2.5%	82 ± 1.0%
	Tablet	29 ± 9.5%	46 ± 2.6%	39 ± 2.3%	43 ± 2.4%
	Technology early adopters	5.7 ± 4.7%	3.2 ± 0.4%	3.0 ± 1.0%	4.6 ± 0.1%
Connectivity	3G	0.0 ± 0.0%	4.0 ± 0.7%	0.0 ± 0.1%	11 ± 0.8%
	4G	35 ± 18%	12 ± 0.3%	28 ± 3.1%	34 ± 0.4%
	WiFi	37 ± 8.9%	57 ± 1.4%	46 ± 2.8%	28 ± 1.0%

For each city, we report the users rate (in percentage) containing each attribute over the total users of an administrative subdivision, averaged over all neighborhoods of the city, along with the standard deviation. Attributes were included only if the corresponding users estimate did not hit the 1.000 users threshold in all districts of a city.

How Facebook determines the audience that corresponds to a specific target is not disclosed in detail by the marketing platform. Some attributes are inferred by Facebook from the self-disclosed information and from the user interactions on the platform. Technology related features are automatically determined by the information collected from the devices used to connect to the platform, which in principle may be more reliable. We queried the marketing API requesting the number of Facebook users aged 13 or above, matching the above targeting characteristics. According to the Meta Ads Manager, Facebook penetration varies across cities and it roughly matches Facebook adoption rates observed at the national level. Facebook users are 56% of the population in Morocco, 72% in Colombia, 69% in the USA, and 81% in Chile (source: https://www.facebook.com/business/tools/ads-manager).

### 3.3. Spatial aggregation of predictive features

As mentioned before, the Facebook marketing API provides audience estimates for a specific geographic area that is specified in each query. Available geographic targeting options vary in each country and our queries used different spatial parameters, depending on the city under study.

In the city of Atlanta, and in general for all US cities, the Facebook marketing API allows to run queries by zip code, that is the spatial granularity for which income data is available. In this case, it was possible to match the predictive features of the audience estimates with the target variable, at the same spatial resolution, without the need of additional data manipulation. In other countries, however, the platform provides fewer geographic targeting options, especially at a high granularity. To overcome this issue, in the case of Bogotá, Santiago, and Casablanca, we run our queries by selecting circles of 1 km radius as our geographic target.

**Figure 2:**
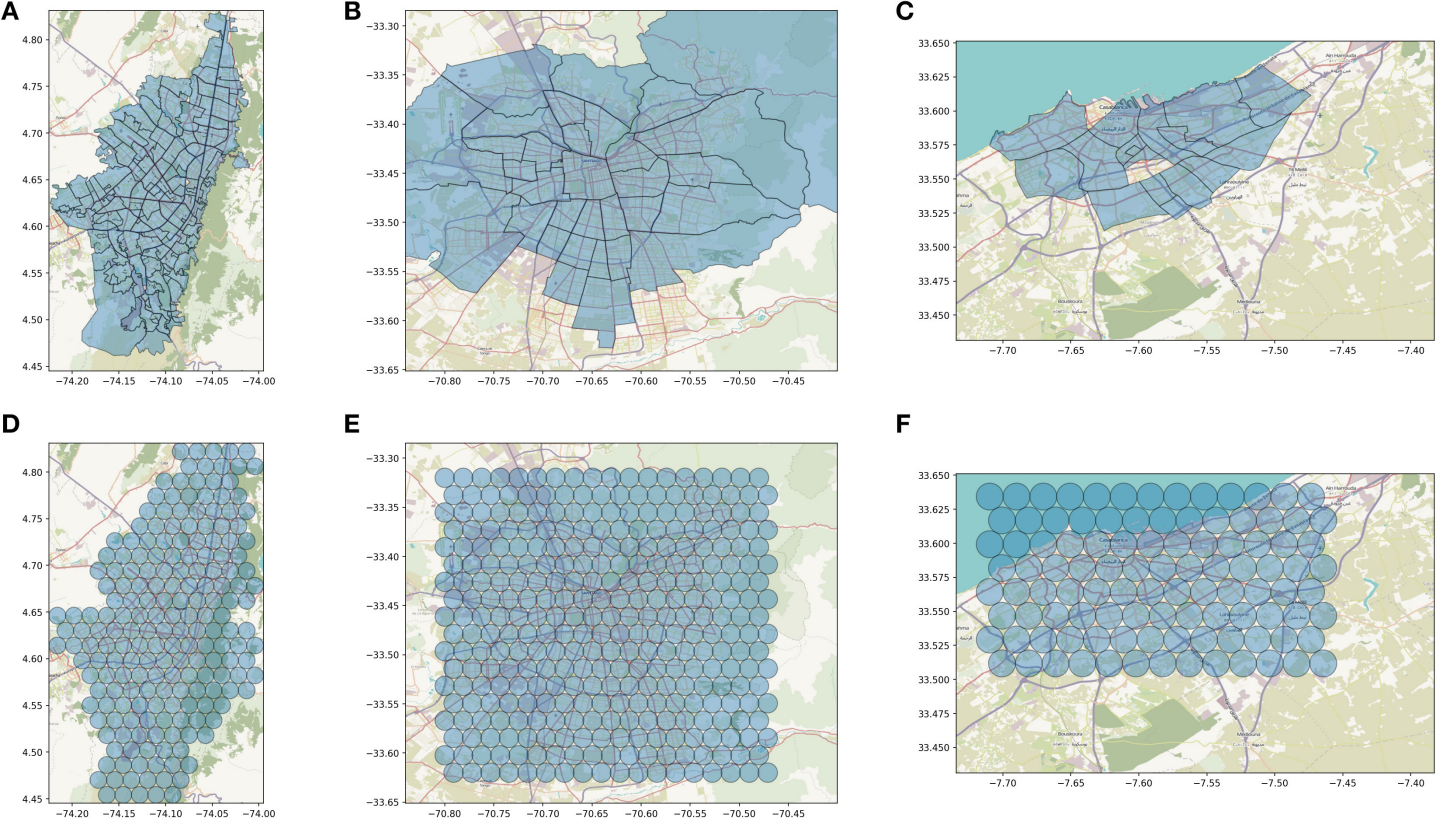
Spatial targeting schemes for the queries of Bogota **(A,D)**, Santiago **(B,E)**, Casablanca **(C,F)**. For each city we show the administrative subdivisions for which socioeconomic data is available (upper panels) and the grid of circles of 1 km radius that we used to collect audience estimates. Only estimates for circles intersecting the administrative units were considered in the models. These are: 196 circles in Bogotá, 324 in Santiago, 104 in Casablanca.


More specifically, we created a grid of equally spaced circles of 1 km radius to cover the area of the three cities as shown in Figure 2 (bottom panels). This corresponds to the highest resolution at which Facebook audience estimates are available from the marketing API. Circles were defined to be densely packed within the bounding box of the city boundaries. We then queried the Facebook API by requesting the number of monthly active users who live in every circle and who match the targeting options defined above. Finally, we projected the audience estimates from the circles to the administrative units as follows. For each circle *i*, and each administrative unit *j* (*comuna*, UPZ or *arrondissement*) we compute the fraction of the area of the circle *i* that intersects the unit *j*, *a*_*ij*_. Then, we compute the estimate of MAU in each unit *j* as the sum of the estimates in the circles intersecting *j*, weighted by the area of intersection:

(1) $$\begin{array}{l} {\text{MAU}}_{j}=\underset{i\in \nu_{j}}{\sum }{\text{MAU}}_{i}a_{ij} \end{array}$$

Where ν_*j*_ is the set of circles intersecting the administrative unit *j*. As a result, in each administrative unit *j*, we obtain an estimate of MAU who match a given targeting option, that we can relate to the SES of that unit.

### 3.4. Classification task

As our SES indicators differ in scale and type, across cities, we describe our problem as a classification task, by training a model to predict the top 50% (or 25 or 75%) administrative units that are more likely to belong to the low (or high) SES class. This can be defined as an effective binary classification problem, where we predict the sequence of labels −1, 1 that are assigned to all districts in a city. More in details, we formally define the problem as follows.

For each city, in each administrative unit, we considered the attribute counts of all Facebook users (aged 13 or more), normalized by the total number of Facebook MAUs as input predictors for a binary classification task. The feature matrix ***X*** ∈ ℝ^*n*×*p*^ is computed as follows: (i) each row corresponds to a district (at the chosen aggregation level) with all normalized Facebook variables greater than zero, and (ii) each column corresponds to a feature without zero entries in every district of the city. Table 2 reports normalized Facebook attribute (in percentage), averaged over all administrative units, included in the input matrix of the classification model for each city.

For each row in ***X*** (i.e. for each district of the city), we assign a binary indicator of SES by choosing as a threshold the *k*-th percentile according to the distribution of the SES indicator in each city: districts with SES indicator above the *k*-th percentile are assigned as 1, and –1 otherwise (except for Casablanca, where districts are assigned as 1 if the poverty rate is below the *k*-th percentile, and –1 otherwise). In this way we obtain a target variable ***y***^(*k*)^ ∈ {−1, 1}, with positive label associated to high income districts across all the considered cities. The percentiles are chosen to be among 25, 50, or 75 and correspond to the quartiles of the distributions.

With the pair (***X***, ***y***^(*k*)^) for each city we train a random forest classifier to predict the socioeconomic indicators ***y***^(*k*)^, according to the *k*-th percentile, from ***X***. We choose a random forest model because it can effectively classify non-linearly separated data and it can be easily combined with SHAP (Lundberg et al., 2018), for interpratibility of results. Hyperparameters of the model are tuned with a 5-fold cross validation, searching for the best number of tree estimators and maximum depth of the trees^6^ that maximize the average ROC-AUC score over 30 different combinations of cross-validation folds. We choose to maximize the ROC-AUC score, and we use ROC-AUC to evaluate the model performance, for three main reasons: (i) ROC-AUC it is invariant to the class prior; (ii) we are mainly interested in evaluating ranking predictions; (iii) we care equally about positive and negative classes. The threshold ROC-AUC = 0.5 characterizes the performance of a random classifier and we compare our results against it, to assess our model's performance.

## 4. Results

In this section we describe the results of the binary classification task defined in Section 3.4. For each city we trained a classifier aimed at identifying neighborhoods whose SES indicator stands above or below a given percentile of the distribution, thus effectively defining a high-SES/low-SES binary score for each neighborhood. We show experiments where “native” classifiers trained on a source city are tested on different target cities to study the domain adaptation performance. Moreover, we analyze the most influential features selected by the native classifiers on the related training cities.

We report in tables the average ROC-AUC scores with standard deviation, calculated in different ways if testing the source model on the same city or in another one. On the diagonal of each table we show the model performance in the same city, with the average and standard deviation of cross-validated scores over 30 different fold split combinations of the best performing model. For the values in the off-diagonal we fix the hyperparameters in each city and train 30 different models with these hyperparameters but different random seed initialization, then we predict the SES indicators of a target city. We report average performance of the models on the target cities.

### 4.1. Classification of socioeconomic status

Table 3 shows the classification performance in terms of ROC-AUC score with classifier trained on city in row and tested on the city in column, with diagonal showing “native” classifiers. All classifiers use the median of the SES distribution as a threshold for the binary labeling. As expected, the scores on the diagonal—when the classifier is trained and tested on the same city—are the highest, ranging from AUC = 0.925 for Santiago to 0.819 in Casablanca. Domain adaptation cases show lower scores, with the highest scores achieved by training on Bogotá and testing on Santiago (0.829), and on Atlanta (0.821). In some other combinations, the performance can be even below 0.500, such as in the case of Casablanca to Atlanta (0.350) and to Bogotá (0.478). Note that here, we assume the SES distribution in each city is roughly the same by splitting the class on the median. Next, we ask whether other definitions of high/low SES in terms of percentile of the distribution may be more appropriate.

**Table 3:** Performance of classification in terms of ROC-AUC score with classifier trained on city in row and tested on the city in column, with diagonal showing “native” classifiers.

**Train/test**	**Atlanta**	**Bogotá**	**Santiago**	**Casablanca**
Atlanta, GA, USA [50]	**0.876 ± 0.026**	0.659 ± 0.073	0.802 ± 0.027	0.512 ± 0.066
Bogotá, Colombia [25]	0.792 ± 0.042	**0.925 ± 0.011**	0.760 ± 0.027	0.470 ± 0.094
Santiago, Chile [75]	0.893 ± 0.018	0.730 ± 0.040	**0.993 ± 0.006**	0.483 ± 0.052
Casablanca, Morocco [25]	0.495 ± 0.044	0.464 ± 0.075	0.600 ± 0.083	**0.917 ± 0.034**

For each city, the percentile of the SES distribution that maximizes the ROC-AUC is shown in square brackets. The bold values indicated to highlight the diagonals.

### 4.2. Sensitivity to high/low SES definition

**Figure 3:**
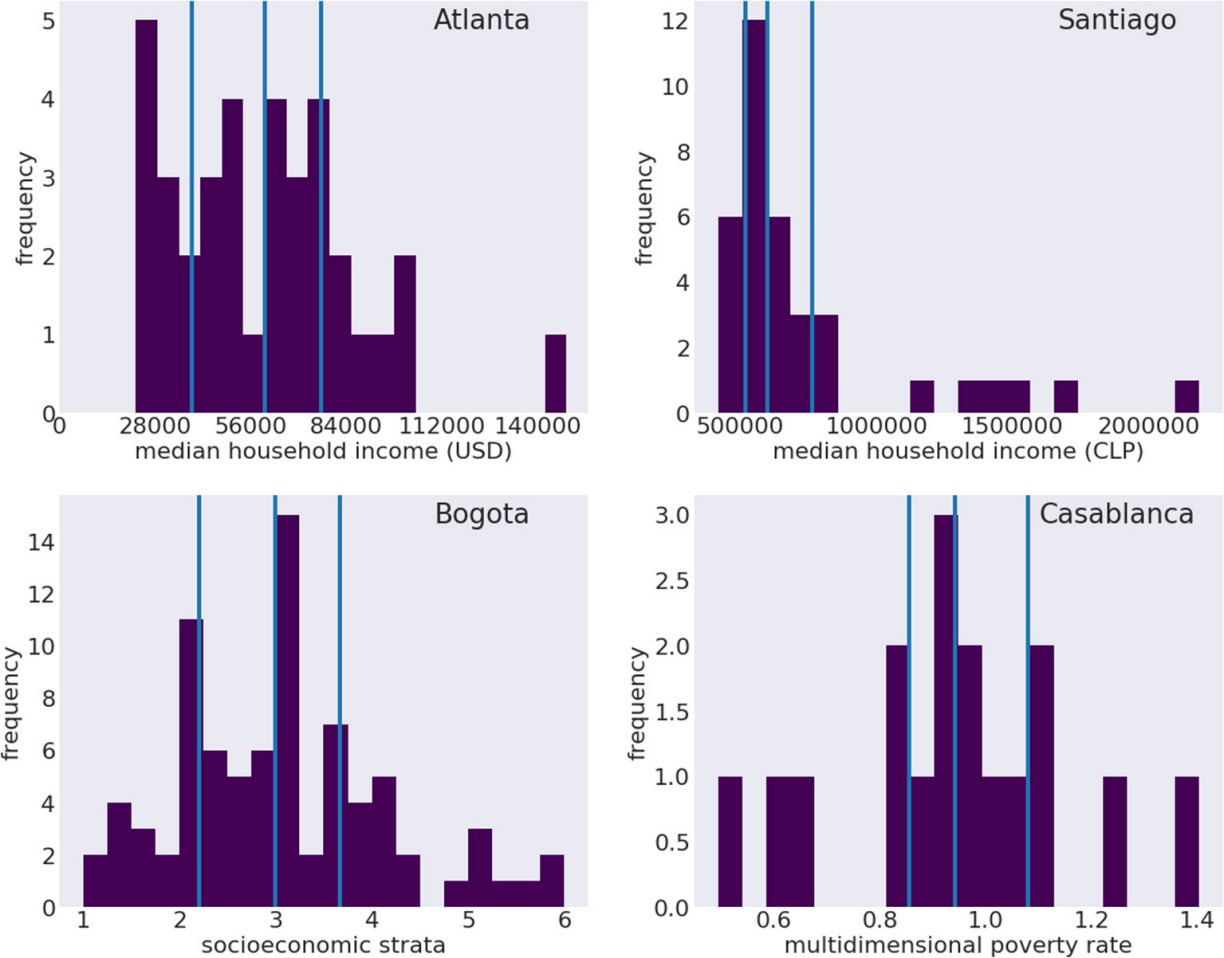
Histograms of socioeconomic indicators distributions in the cities of Atlanta, Santiago, Bogotá and Casablanca. From top to bottom: median household income in Atlanta, USA, by zip code; median household income in Santiago, Chile, by comuna; socioeconomic strata in Bogotá, Colombia, by UPZ; multidimensional poverty rate, in Casablanca, Morocco, by arrondissement. Vertical solid lines indicate to the quartiles of the distributions.

Figure 3 shows the histograms of the SES indicators for each city. Note that while those for Bogotá and Casablanca have been standardized as scores, Atlanta and Santiago data are raw income values, which have a skewed distribution (as is well-known in the literature Benhabib and Bisin, 2018). Since the shape of these distribution is quite different, we ask whether a different threshold to define high/low SES may be more applicable to each location.

Table 3 shows similar setup as Table 4, except the SES thresholds are maximized over the ROC-AUC score of the training city (indicated in brackets of the training city). This optimization results in a lower threshold for Bogotá and Casablanca (25) and a higher one for Santiago (75). We find a limited improvement on the diagonal, with small improvements for Santiago (7% improvement) and Casablanca (10%). However, we find a mixed result for the domain adaptation, with improvements for most cases, most notably Santiago to Atlanta (0.698–0.893), but marked decreases for Bogotá to Casablanca (0.550–0.470) and to Santiago to Casablanca (0.541–0.483), in latter case dropping performance below baseline. Thus, we find that the optimization on the training set may improve the native city performance, but may have unpredictable effect on the performance on other locales. It may be the case that adjusting this threshold should happen separately for the training and testing city, as each may have a different SES distribution.

**Table 4:** Performance of classification in terms of ROC-AUC score (± standard deviation) with classifier trained on city in row and tested on the city in column, with diagonal showing “native” classifiers.

**Train/test**	**Atlanta**	**Bogotá**	**Santiago**	**Casablanca**
Atlanta, GA, USA	**0.876 ± 0.026**	0.659 ± 0.073	0.802 ± 0.027	0.512 ± 0.066
Bogotá, Colombia	0.821 ± 0.020	**0.924 ± 0.007**	0.829 ± 0.027	0.550 ± 0.078
Santiago, Chile	0.698 ± 0.060	0.711 ± 0.068	**0.925 ± 0.015**	0.541 ± 0.078
Casablanca, Morocco	0.350 ± 0.078	0.478 ± 0.061	0.555 ± 0.121	**0.819 ± 0.030**

All classifiers use the median of the distribution to define a high/low SES threshold. The bold values indicated to highlight the diagonals.

Table 5 shows the performance of classifiers trained on different thresholds (25th, 50th, and 75th percentiles), and applied to the testing set using the same threshold. We find that some of the results are very sensitive to the threshold, for instance Atlanta to Santiago performs at AUC = 0.802 to 0.580 from threshold 50 to 25, respectively. The performance also can range widely within the same city, such as in the case of Casablanca, which performs at AUC = 0.917 with threshold 25, and at 0.573 with threshold 75. For Casablanca to Bogotá, the scores on domain adaptation do not pass 0.500, at any threshold. In fact, any adaptation from Casablanca seems to produce poor results (except perhaps to Santiago at threshold 75), possibly due to the fewer number of sub-regions (making spatial aggregation imprecise), compared to the other cities. We conclude that the performance of the model can be sensitive to the definition of what is high and low SES, especially when adapting models between cities, necessitating thorough testing at different thresholds.

**Table 5:** Performance of classification in terms of ROC-AUC (± standard deviation) with classifier trained on city in row and tested on the city in column, with diagonal showing “native” classifiers.

**Train/test**	**Atlanta**	**Bogota**	**Santiago**	**Casablanca**
Atlanta	0.869 ± 0.027	0.520 ± 0.165	0.580 ± 0.131	0.430 ± 0.113
	0.876 ± 0.026	0.659 ± 0.073	0.802 ± 0.027	0.512 ± 0.066
	0.768 ± 0.048	0.514 ± 0.105	0.793 ± 0.165	0.488 ± 0.051
Bogota	0.792 ± 0.042	0.925 ± 0.011	0.760 ± 0.027	0.470 ± 0.094
	0.821 ± 0.020	0.924 ± 0.007	0.829 ± 0.027	0.550 ± 0.078
	0.827 ± 0.028	0.906 ± 0.011	0.632 ± 0.055	0.321 ± 0.074
Santiago	0.689 ± 0.038	0.695 ± 0.073	0.740 ± 0.041	0.459 ± 0.097
	0.698 ± 0.060	0.711 ± 0.068	0.925 ± 0.015	0.541 ± 0.078
	0.893 ± 0.018	0.730 ± 0.040	0.993 ± 0.006	0.483 ± 0.052
Casablanca	0.495 ± 0.044	0.464 ± 0.075	0.600 ± 0.083	0.917 ± 0.034
	0.350 ± 0.078	0.478 ± 0.061	0.555 ± 0.121	0.819 ± 0.030
	0.604 ± 0.043	0.358 ± 0.026	0.812 ± 0.090	0.573 ± 0.053

For each city, three different values of the ROC-AUC are shown, corresponding to different high/low SES thresholds applied to both train and test: 25th, 50th, and 75th percentiles.

### 4.3. Feature importance

To explore the importance of various Facebook advertising features in our classification model, we examine the SHAP (SHapley Additive exPlanations) values associated with each, in the four cities (Lundberg and Lee, 2017). SHAP is a method to explain model predictions based on Shapley Values from game theory. In particular, we use TreeSHAP (Lundberg et al., 2018), an algorithm to compute SHAP values for tree ensemble models, such as the random forest classifier of our study.

**Figure 4:**
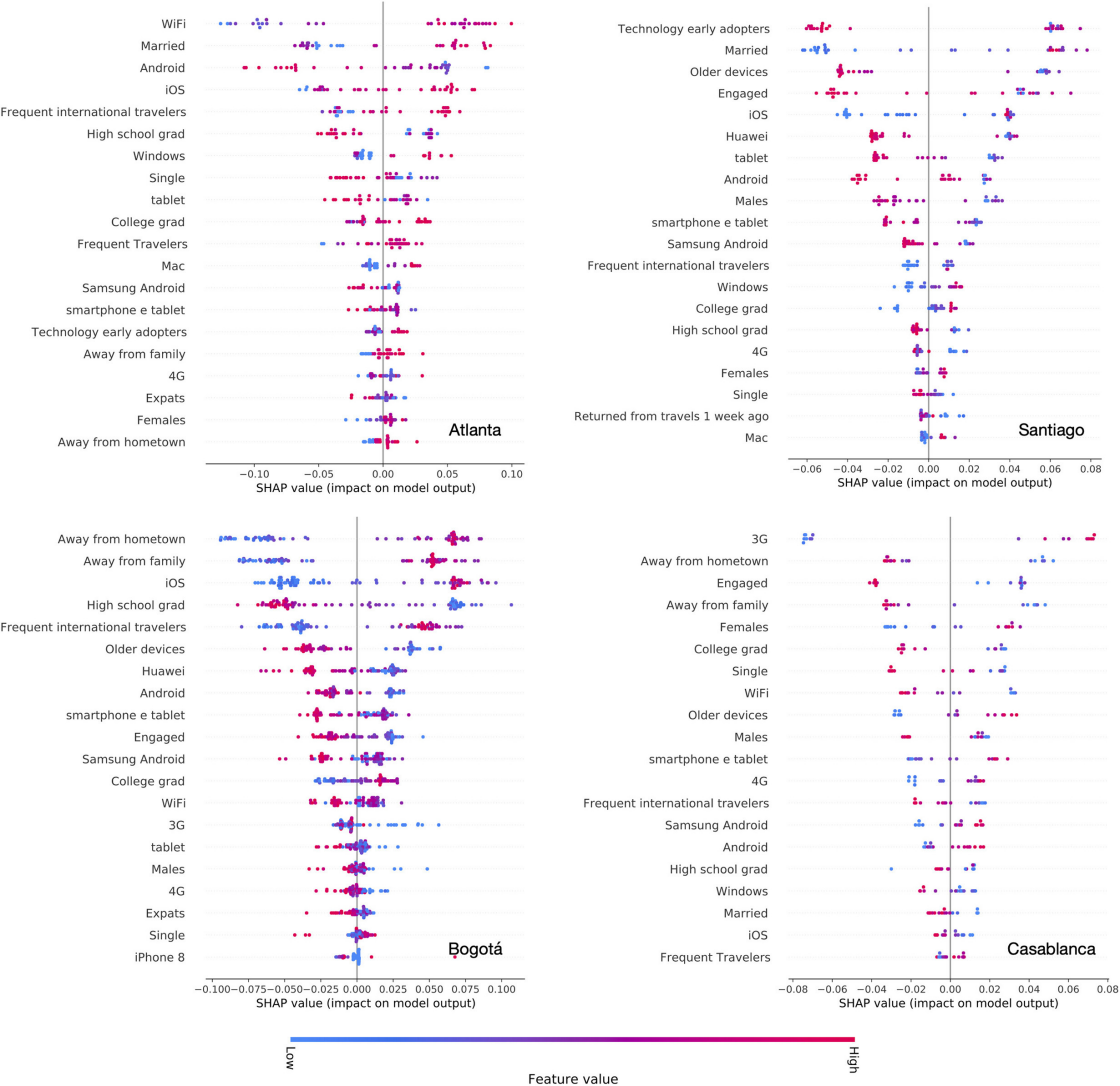
SHAP plots of feature importance. For each city, we plot a summary of the effects of the top 20 features, ranked by their importance. Color represents the feature value (blue is low and red is high). The SHAP value, on the horizontal axis, indicates the feature importance on the model output, with larger values corresponding to higher relevance.

Figure 4 displays a summary of feature importance in the “native” classification model for the four cities under study, assuming the median of the SES distribution to discriminate between high and low SES. For each feature, the distribution of their SHAP values is shown on the horizontal axis, indicating the impact on model output. Color code describes the feature value, with red indicating higher values, and blue corresponding to lower.

Interestingly, features that are most predictive in the four cities vary, depending on the urban setting. In general, technology-related features are important predictors in all settings, as found in previous studies (Fatehkia et al., 2020b). However, specific features that are most important vary by city. The fraction of iOS users is among the top predictors of wealth in Atlanta, Santiago and Bogotá but not in Casablanca, where instead the use of 3G/4G and Android adoption appear to be the most predictive feature (higher share of 3G and Android is associated with higher SES). On the contrary, the adoption of Android, Android based smartphones, or older devices is associated with lower wealth.

Beside the adoption of certain technologies of products, features related to interests or socio-demographic characteristics are often highly predictive of SES. This is the case of education levels, where the distinction between “High school graduates” and “College education” provides an important signal to identify wealthier neighborhoods, in Atlanta, Bogotá, and Santiago. Travel related features are also significant predictor of high SES. This is observed in Atlanta, where a higher presence of “Frequent international travelers” and “Frequent travelers” predicts higher SES of a zip code. In Bogotá, a higher presence of users who are “Away from hometown” and “Away from family” strongly predicts higher SES of a neighborhood. A similar but much weaker signal is observed in Atlanta. Marital status appears to be a strongly predictive feature—ranked 2nd by SHAP—in Atlanta and Santiago. A higher fraction of married users is associated with higher SES. On the contrary, a higher proportion of “Engaged” users is associated with lower SES, in Bogotá and Casablanca. Finally, gender is also associated with SES. A higher presence of female users in a neighborhood is predictive of higher SES in Casablanca, and similarly in Santiago and Atlanta.

Overall, with respect to the other cities, Casablanca stands out as an outlier with respect to the features that are associated with SES and the relative direction of association. For instance, in Casablanca, we could not identify a signal from education levels or travel behavior that was coherent with the results in Atlanta, Santiago and Bogotá. Such discrepancy may explain the poor transferability of the classifier trained on data from Casablanca to other cities, and vice versa, as shown in Table 3. Casablanca is also the city with the smallest number of data points in our study, and with the smallest area covered by the municipality. This could affect the spatial targeting scheme we adopted and result in a less accurate mapping of users' characteristics across neighborhoods.

## 5. Discussion and conclusion

The present study proves the feasibility of modeling socioeconomic inequality at a high spatial resolution using aggregate statistics collected by a social media platform. For four case studies, we show that we are able to build highly accurate models (with AUC scores over 0.90) that distinguish locales which have higher and lower SES. Such models may be important in monitoring changes in the neighborhood economy, especially with local changes due to economic, social, or environmental causes. Because standard measures can have lags of months or years (such as a population census), social media may provide a more up-to-date estimate to supplement surveys. However, Facebook's user statistics may also be updated at some internal schedule, and more work is necessary to evaluate the dynamism of the statistics it provides and whether or not such data can indeed be used to reliably fill temporal gaps in census surveys. For instance, recent work (Kondmann and Zhu, 2020) has shown that monitoring SES changes over time with novel data streams can be challenging. Already, Facebook Ads have been used to monitor highly volatile situations, in the context of disasters and emergencies, such as the exodus of Venezuelans during the 2018–2019 crisis (Palotti et al., 2020). Further, some population behaviors, such as technology use, may be updated in the platform at a faster rate. Such attribute is the brand and make of the phones logging into Facebook (which was used to estimate gender gaps in tech use by Sabri et al., 2021). As weather-related disasters become more common, putting urban populations in strenuous circumstances due to environmental degradation, sea level rise (France24, 2021), and fires (UN Environment Programme, 2022), capturing up-to-date signals around the economic impact of vulnerable populations is paramount to timely intervention.

Adaptation of such models from different locales, however, proved to be difficult, as we have shown with a selection of countries from three continents. Not only do models trained on a different country vary widely in performance (from up to AUC around 0.80 to same as random), this performance may change drastically if in the target city the distinction between high vs. low SES separation changes. For instance, a model trained on Atlanta at 25th percentile SES threshold performs fairly well in Atlanta (AUC = 0.869), but is achieves only AUC = 0.580 when applied to Santiago at the same threshold. Instead, it performs best at threshold at the 75th percentile. Future research into the calibration of a more sophisticated model with the information of the SES distribution of the source and destination locales is necessary to fully take advantage of the available data.

The importance of the technological signals in the models built, both for the cities in the developing countries and developed, highlights the ongoing burden of technological inequality. Digital inequality has been found to be related to the demographic attributes, traditionally affecting those in rural settings, but also importantly correlating with education levels and unemployment (Blank et al., 2018). Low access to or utilization of information and communication technologies (ICT) may also result in the exclusion from transportation services, especially as ICT becomes increasingly integrated into public services (Durand et al., 2022). Ongoing efforts for tracking technology access, such as the Australian Digital Inclusion Index (ADII) (Wilson et al., 2019), are necessary to monitor the access to technology, and to ensure that the relevant principles adopted at the World Summit on the Information Society (convened by the United Nations in Geneva in 2003) are put into practice (World Summit on the Information Society, 2003).

The feature exploration also points to the fine distinction between the concept of an “international traveler” (associated positively with SES in Atlanta) and those living “away from hometown” (negatively associated with SES in Casablanca). Mobility, including that for economic purposes, has been shown to be an important indicator around SES (Lenormand et al., 2015; Millanida Hilman et al., 2021; Moro et al., 2021; Macedo et al., 2022).

This data source has several advantages over the traditional survey methods. First, it is publicly available and it is possible to gather large amounts of data *via* the website's API. Second, it is updated regularly, and may provide a more up-to-date view of the situation than an expensive census or survey. Third, disaggregation by gender and age provide a way to focus on target demographics of interest, such as in previous work on gender inequality in India (Mejova et al., 2018). Fourth, because the individual data is not released by Facebook, this resource allows the study of populations without the compromise of privacy of any captured individuals. Fifth, this data source may reveal populations which are not officially counted by the local authorities, or who are temporarily passing through the area, such as recent study of the Venezuelan migration into Colombia (Palotti et al., 2020). Finally, it is possible to explore the demographic, behavioral, and technological correlates of socioeconomic index in each urban setting. For instance, in previous study of the Italian municipalities, those with lower income had a higher interest in cooking, restaurants, and gambling (Rama et al., 2020). Although not explored in this work, health-related interests may also help identify areas of need (Araujo et al., 2017). In our case study, we provide an analysis of four cities of varying SES dynamics, Facebook penetration, and part of the world. We illustrate that the signals provided by Facebook advertising platform are indeed related to socioeconomic indicators, and in fact may provide a finer-grained detail on the separation of their inhabitants by SES.

As a comparison, several alternative data sources have been proved to be effective to measure economic development. In particular, thanks to the recent advances in image processing and machine learning, information extracted from satellite areal imagery represents one of the most widely investigated resource to map SES at different scales (Jean et al., 2016; Burke et al., 2021; Chi et al., 2022). Satellite-based measurements can achieve a very high predictive accuracy combined with a high spatial resolution. However, such levels of accuracy come at a significant financial cost since high-resolution (< 1*m*) satellite imagery must be purchased from private providers and it is usually expensive. Also, satellite-based measurements of development often lack interpretability and such issue has been addressed only recently (Abitbol and Karsai, 2020; Ayush et al., 2020). Compared to satellite images, social media advertising data are usually less expensive to collect, and their relationships with SES are easier to interpret. Also, social media data may be more suitable to capture socio-demographic changes that may reflect changes of SES on short timescales. Combining the two data sources, satellite imagery and advertising data, may provide complementary information to advance SES mapping at a high granularity.

The above advantages come with marked limitations, which must be addressed when utilizing this data source. The dynamic nature of this data reminds us that Facebook may update it based on the internal scheduling and needs of the company, and it is not certain just how current the estimates are. Further, the black-box nature of the tool puts in question whether identification of individuals in various categories performs uniformly across locales. For instance, whether the gender classification (when such information is not provided by the user) works equally well for African, Asian, and Middle-Eastern users as it does for English-language ones is questionable, given the known biases of “minority language” NLP systems (Blodgett et al., 2020). However, if we study fairly homogeneous populations within each locale, the analytical pipeline applied to the users will hopefully not be as subject to such bias as a comparative study across countries or language groups. Another source of bias may come from the internal benefit to the company to find particular users that are highly sought-after by the advertisers, such as those having the funds to spend on the advertised product, or having the demographics matching the advertised messages.

Beyond the limitations of the data source, this study in particular has notable shortcomings, some of which present interesting future research directions. We present only one way of using the 1 km radius circles to survey an area, but other packing and aggregation methods may be possible. For example, when projecting from circles to area, the geographic overlap may be enriched by the population data of the two areas. The fact that for two cities we use SES metrics that are only related to household income, instead of the median household income, limits the extent of our conclusions regarding model's domain adaptation. However, income plays a prominent role in these scores, making them likely comparable. This limitation reflects the challenges of working with SE data produced by different governments, necessitating greater international collaboration in the fight on poverty. Further, it is difficult to make generalizations based on 4 cities. Future research work should focus on investigating the transferability of poverty targeting models across domains, from high-income to low-income economies, in several cities. Unfortunately, the public availability of the fine-grained and standardized SES ground truth data is often the limiting factor.

In this study, we modeled socioeconomic indicators across four cities situated in different continents and undergoing different economic development stage. We showed that, using Facebook advertising estimates, it is possible to obtain fine-grained models of SES of populations in the urban areas of Atlanta, Santiago, Bogotá, and Casablanca. For each city, we show that a different set of demographic, technological and behavioral variables may be associated with SES.

Using methodology proposed here, we hope that the SDG goal of poverty reduction will be monitored at a fine spatial resolution in the urban areas worldwide, both to gauge the improvements in socioeconomic factors, and to better understand the multiple dimensions of wellbeing. As we continue to build such systems, we encourage researchers and policy-makers to continue experimentation with this near-real-time, fine-grained data source, especially in the dynamic urban environments of the developing world.

## Data availability statement

The datasets presented in this study can be found in online repositories. The names of the repository/repositories and accession number(s) can be found below: https://github.com/simonepiaggesi/predicting-city-poverty-facebook.

## Author contributions

SP and SG collected and processed the data, and performed the analysis. SP, MK, YM, AP, and MT designed the study, interpreted the results, and wrote the manuscript. All authors read, commented, and approved the final version of the manuscript.

## Funding

SG, SP, YM, AP, and MT gratefully acknowledge the support of the Lagrange Program of the ISI Foundation funded by CRT Foundation. MK acknowledges to participate as the Fellow of the ISI Foundation and support from the H2020 SoBigData++ project (H2020-871042) and the DataRedux ANR project (ANR-19-CE46-0008). AP acknowledges partial support from Intesa Sanpaolo Innovation Center. The funders had no role in study design, data collection and analysis, decision to publish, or preparation of the manuscript.

## Conflict of interest

The authors declare that the research was conducted in the absence of any commercial or financial relationships that could be construed as a potential conflict of interest.

## Publisher's note

All claims expressed in this article are solely those of the authors and do not necessarily represent those of their affiliated organizations, or those of the publisher, the editors and the reviewers. Any product that may be evaluated in this article, or claim that may be made by its manufacturer, is not guaranteed or endorsed by the publisher.

## Footnotes

^1^ https://sdgs.un.org/goals

^2^ https://www.worldbank.org/en/topic/measuringpoverty

^3^ https://developers.facebook.com/docs/marketing-apis/

^4^ https://developers.facebook.com/docs/marketing-api/reference/ad-campaign-delivery-estimate/

^5^ https://github.com/facebook/facebook-python-business-sdk

^6^n_estimators ∈ {10, 50, 100, 200, 300} and max_depth ∈ {2, 5, 20, 50, 100}.
